# Association between Intimate Partner Violence and Abortion in Nepal: A Pooled Analysis of Nepal Demographic and Health Surveys (2011 and 2016)

**DOI:** 10.1155/2020/5487164

**Published:** 2020-08-31

**Authors:** Dipendra Singh Thakuri, Pramesh Raj Ghimire, Samikshya Poudel, Resham Bahadur Khatri

**Affiliations:** ^1^Health and Nutrition Department, Save the Children, Surkhet, Nepal; ^2^Ujyalo Nepal, Ratnanagar Municipality, Chitwan, Nepal; ^3^School of Public Health, Faculty of Medicine, University of Queensland, Brisbane, Australia

## Abstract

**Background:**

Intimate partner violence (IPV) adversely affects female reproductive health in different ways. However, the relationship between IPV and abortion has not been adequately examined in Nepal. This study is aimed at examining the association between IPV and abortion in Nepal.

**Methods:**

Data for this study was derived from the Nepal Demographic Health Surveys (NDHS) of 2011 and 2016. A total of 8641 women aged 15-49 years were selected for the violence module in NDHS 2011 and 2016. The analysis was restricted to 2978 women who reported at least one pregnancy five years preceding each survey. Among them, 839 women who experienced different forms of violence were included in the analysis. Various forms of IPV were taken as exposure variables while abortion as an outcome of interest. The study employed logistic regression analysis to examine the association between IPV and abortion.

**Results:**

Nearly one in three (28.2%) women experienced any forms of IPV. A total of 22.2% women experienced physical violence. Almost one in five (19.5%) women were slapped. More than half (52.8%) of the women with no education experienced IPV. The logistic regression analyses showed a significant association between IPV and abortion. Women with severe physical violence had nearly two-fold higher odds (adjusted Odds Ratio (aOR) = 1.68; 95% CI: 1.06, 2.64) of having abortion. Similarly, women who reported physical violence were more likely to have abortion (aOR = 1.54; 95% CI: 1.09, 2.19) compared to those who did not experience such violence.

**Conclusion:**

Intimate partner violence is associated with abortion in Nepal. It is imperative that effective implementation of IPV-preventive measures through the promotion of appropriate social and policy actions can help reduce abortion in Nepal.

## 1. Introduction

Intimate partner violence (IPV) is defined as any behaviour within an intimate relationship that causes physical, sexual, and psychological harm [1]. These behaviours include but not limited to the act of physical aggression, sexual coercion, and psychological abuse [1]. Violence against women is a serious public health problem [2–5]; it is considered as a violation of human rights [6, 7]. It is evident that IPV during pregnancy places women in a greater vulnerable situation and has been documented as an important contributor to adverse maternal and fetal health outcomes [8]. Adverse maternal health outcomes include unintended pregnancy, induced abortion, miscarriage, pregnancy complications, hypertension, delivering low birth weight, physical injuries, stress, and lack of fertility control and personal autonomy [4, 6, 7, 9–11]. Studies conducted in Bangladesh found that women in violent relationships are more likely to report miscarriage and stillbirth [7, 12]. A multicountry review study found that 30% of the partnered woman (aged 15–69 years) experienced physical or sexual violence in their lifetime [13].

In Nepal, violence against women is common that 51.9% of young women experience physical and sexual violence in their lifetime [14]. Nepal is a patriarchal society, and gender-based discrimination is highly prevalent [15–17]. In a patriarchal culture, men tend to have higher control over women decision making and health seeking practices and women lack control of resources. More than two-fifth (44%) of men agreed that women deserve to be beaten while 21% of men and 23% of women believed that husband beating spouses is justified if wives refuse to have sex on demands [2, 18]. A global multicountry review study has reported that almost half of the women globally suffer physical abuse from their partner [14]. Past evidence from Nepal showed a higher proportion of physical violence during reproductive age [19]. A study conducted in four districts of Nepal showed that nearly three in five women (59.2%) ever experienced some forms of violence from their partner [20]. A hospital-based study conducted among 950 urban pregnant women in the capital city of Nepal revealed that 33% of women experienced psychological and physical violence and sexual abuse during pregnancy [8].

Literature suggests that women in a violent relationship may have limited or no control over mutual sexual intercourse [12, 21]. It is urged that controlling behaviour of partner can restrict women from accessing the family planning methods [21, 22], which may lead to unwanted pregnancies and abortion [23]. Evidence also suggested that abortion was associated with the experience of IPV among women [12, 24], so as the higher rate of unintended pregnancy [25]. A study in Bangladesh revealed higher rates of unintended pregnancy among women who experienced severe forms of physical violence [26]. Studies conducted in China and the USA reported that most abortions end with unintended pregnancies, which mainly result from ineffective use and/or nonuse of contraceptives [27–30] and rape being the leading cause of pregnancy termination [1].

In Nepal, abortion is considered to be the third leading cause of maternal death [31]. Nepal legalised abortion in 2002, and both the public and the private sectors started providing abortion services across the country since 2004 [31]. The common reasons for abortion as reported in Nepalese literature are women's knowledge of abortion legality, maternal and fetal health risk, lack of desire for children, child spacing, child sex, and low income [29, 31–33]. Studies from countries in South Asia, primarily in Bangladesh [34], India [35], and Pakistan [36] have established an association between IPV and unwanted pregnancies. However, there is a lack of clarification on the impact of IPV with abortion [34, 37].

A large body of literature has examined and established the relationship between IPV and abortion, especially in low- and middle-income countries (LMICs). Despite the availability of nationally representative data from the NDHS, there have been limited studies conducted to examine the association between IPV and abortion in Nepal. This study is aimed at estimating the prevalence of intimate partner violence and examining the association between IPV and abortion. Findings from this study are expected to inform policymakers, program managers, and public health professionals to design policies and programmatic strategies on abortion services in Nepal.

## 2. Methods

### 2.1. Study Design and Sampling

The present study used nationally representative survey data from the Nepal Demographic and Health Survey (NDHS) 2011 and 2016 (available from https://dhsprogram.com/data/available-datasets.cfm). Detailed sampling methodology is provided in reports of NDHS 2011 [19] and 2016 [17]. In brief, NDHS used multistage cluster sampling design and collected data on several sociodemographic and health indicators, including IPV, and abortion. The average response rate of NDHS 2011 and 2016 was 98%. From NDHS 2011(*N* = 4197) and 2016 (*N* = 4444) data, a total of 8641 women aged 15–49 years who were selected to participate in the violence module survey were merged for this study. The violence module of the survey consists of questions on domestic violence that was administered in the subsample of households. Only one eligible woman per household was randomly selected for the module. Special sampling weights were used to adjust for the selection of only one woman per household and to ensure that the violence subsample was nationally representative [17, 19].

**Figure 1:**
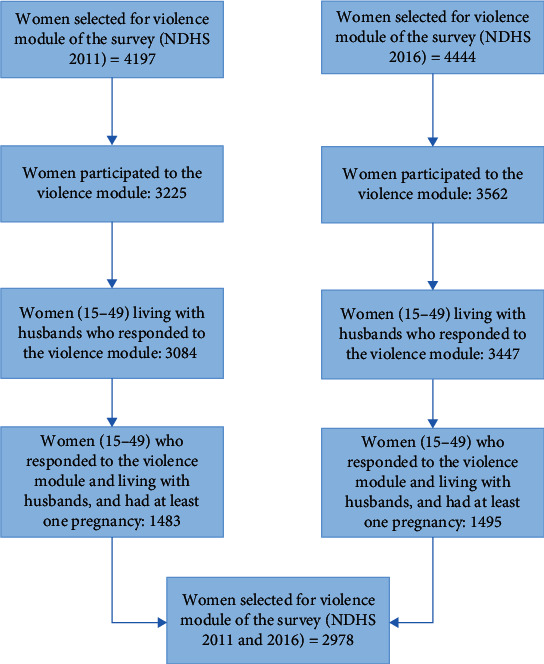
Participant selection process for the violence module survey in the NDHS 2011 and 2016.

Figure 1 shows the participants included for this study. A total of 8641 women were selected from the NDHS 2011 and 2016. About 78% of the total selected women participated and responded to the violence module, which included 3225 women in 2011 and 3562 in 2016. Furthermore, excluding unmarried and divorced, 6531 married women aged 15-49 years responded to the violence module in both surveys. Moreover, one-third of the total selected women (2978) were currently married and living with a husband and had at least one pregnancy during the study period (Figure 1).

### 2.2. Outcome Variable

In NDHS 2011 and 2016, women were asked to report all pregnancy outcomes (live birth, stillbirth, miscarriage, and abortion) five years prior to each survey. To capture pregnancy that resulted in abortion, women were further asked if something was done to terminate the pregnancy [17, 19]. The outcome variable of this study was abortion coded as “1” (if something was done to end the pregnancy) and “0” (otherwise) [17, 19].

### 2.3. Exposure Variables

The exposure variables were women exposed to any form of physical, sexual, and emotional violence by their partner. In NDHS, different types of violence were measured by asking a series of questions. If women were hit, slapped, kicked, or done something else to hurt by husband/partner were considered as physical violence. If women were forced by threats or in any other way to have sexual intercourse or to perform any other sexual acts or they did not want to do so were considered as sexual violence. Similarly, if the respondents had ever been humiliated, threatened with harm and insulted, or made to feel bad by husband/intimate partner were categorised as emotional violence. Specifically, any physical violence included any of the following behaviours from a husband/partner: (i) pushing, shaking, or throwing something at her; (ii) slapping her; (iii) twisting an arm or pulling her hair; (vii) punching with partner's fist or with something that could hurt her; (v) kicking her, dragging her, or beating her up; (vi) choking or burning; and (vii) threatening or attacking with a knife or other weapon. The physical violence in original data files of NDHS 2011 (NPIR60FL) and 2016 (NPIR7HFL) has been categorized as less severe and severe. Any less severe physical violence included (i) pushing, shaking, and throwing something at female partner; (ii) slapping; (iii) twisting arm or pulling hair; or (iv) punching with partner's fist or with something that could hurt her; any severe physical violence included (i) kicking, dragging, or beating up; (ii) choking or burning; or (ii) threatening or attacking her a knife or other weapon.

### 2.4. Covariates

Covariates were selected based on previous studies [31, 38, 39] and the available information in the NDHS 2011 [19] and 2016 [17], including wealth status, ethnicity, age of respondents, level of education, women's empowerment in decision making, and intimate partner's addiction of alcohol consumption. Variables such as ethnicity, age and education status were further categorised for this study. The Government of Nepal has categorised ethnicity into six groups Dalit (Hill and Terai), Janajati (Indigenous Hill and Terai caste group), Madhesi (non-Dalit Terai caste groups), religious minorities (Muslims), upper-caste groups (Brahman/Chhetri), and Others (Thakuri and Sanayshi). However, socioeconomic and geographical similarities [38, 40] in ethnicity was categorised into four groups: Brahman/Chhetri, Janajati, Dalit, and Others, including Muslims and Madhesi. Respondent's age was categorised into three groups: 15-24 years, 25-34 years, and 35-49 years. The education level for respondents and their husbands/partners were categorised into four groups: none (no education), primary (1-5 grades), secondary (9-10 grades), and higher (year 11 or above). The number of living children as reported by women was categorised into four groups: none, 1 child, 2 children, and three or more children. The ecological region was categorised as (1) Mountain, (2) Hill, and (3) Terai. The NDHS applied an asset-based approach to estimate household wealth quintile, and household wealth status was categorized as poorest, poorer, middle, richer, and richest [41]. In regard to women's decision making power on their own health care (whether women participated in at least one of the decisions regarding their own health care, major household purchases or visits to their family or relatives) [42], it was categorised into three groups: involvement of husband and other members, involvement of husband and women, and involvement of respondent alone. Husband/partner's alcohol drinking habit was categorised as yes or no.

### 2.5. Conceptual Framework

**Figure 2:**
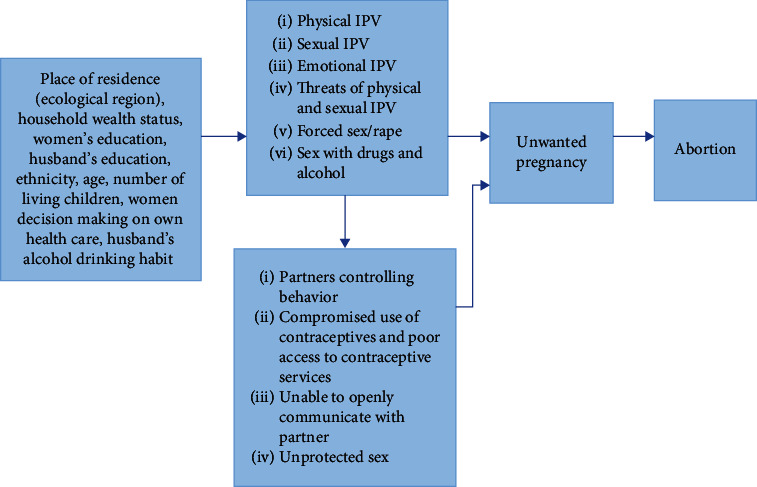
The conceptual framework of intimate partner violence and abortion adapted and modified 207 from Azevedo et al. 2013 [11].

Figure 2 illustrates the relationship between violence and abortion. For this study, we adopted and revised the conceptual framework developed by Azevedo and colleagues (2013) (Figure 2). The framework comprises of sociodemographic characteristics of women, characteristics of partner, and other risk factors. The framework guides this study to conceptualize the relationship between IPV and abortion (Figure 2).

### 2.6. Statistical Analysis

Data were analysed using STATA (version 14.1) (Stata Crop, Texas, USA). In the descriptive analyses, the characteristics of the study participants were presented in the form of frequency number and the proportion (%) with 95% confidence intervals (CI). Logistic regression models were used to examine the association between each exposure variable (different forms of IPV) and abortion. Survey function “svy” in STATA was applied to adjust for sampling weight and clustering effect, and *p* value < 0.05 was considered a significant level.

### 2.7. Research Ethics

This study used publicly available secondary data derived from the NDHS 2011 and 2016. These surveys were approved by an ethical review board of ICF Marco International, Maryland, USA, as well as from the Nepal Health Research Council. The first author got permission from DHS program (USA) to use those datasets for this study. The DHS data are publicly available and accessible at https://dhsprogram.com/data/available-datasets.cfm on online request.

## 3. Results

Table 1 shows the different forms of IPV. A total of 28.2% of women experienced any forms of IPV. More than one in five (22.2%) women experienced physical violence. Nearly one-fifth of women (19.5%) were slapped, and 13.6% were pushed and shook by their partner. Over one in ten (11.2%) women experienced sexual violence. Additionally, one in five (21.8%) women experienced less severe physical violence followed by emotional violence (14.0%). Almost one in 10 (9.4%) women experienced any severe physical violence (Table 1).

Table 2 presents the prevalence of different forms of IPV and abortion five years preceding the survey. Over a quarter (28.2%) of women reported having ever experienced any form of IPV. One in ten (10.2%) women reported abortion (Table 2).

Table 3 shows the sociodemographic characteristics of the participants (*n* = 839) included in this study. Women experiencing IPV varied from 2.6% (women having no living children) to 59.3% (women who live in Terai region). Over a quarter (28.2%) of women experienced any forms of IPV. Of 839 women who experienced violence, more than half (52.8%) of the women with no education experienced IPV in their pregnancy. More than two in five women (44.6%) with IPV were from women whose husband educated secondary or higher-level, and 48.5% of women were aged 25-34 years. A quarter of women from the middle-wealth quintile and 3 in 10 Janajati (indigenous groups) women experienced IPV. Nearly half (46.6%) with a husband with a history of alcohol use and just over six in 10 women having husband and other members involved in decision making for her own health experienced IPV (Table 3).

Table 4 shows abortion among women with and without IPV. Overall, almost 1 in 10 (9.8%) women had abortion. Just over one-tenth (10.2%) women who experienced IPV had abortion. Nearly two in five (39.3%) of the women with two living children experienced violence and had abortions. Furthermore, more than half (56.0%) of the women with secondary or higher education who experienced IPV had abortions. Less than half (44.7%) of the women involved in decision making of her own health with her husband experienced violence and had abortion. Over 6 in 10 (62.3%) of women with a history of husband's alcohol use who experienced IPV had abortions (Table 4).

Table 5 shows the association between various forms of intimate partner violence and the likelihood of abortion. In multivariate logistic regression analysis, a significant association was found in IPV and the risk of abortion. Women ever experiencing any severe violence had the strongest association (aOR = 1.68; 95% CI: 1.06, 2.64) with abortion. Women who experienced physical violence were also more likely to have abortion (aOR = 1.54; 95% CI: 1.09, 2.19) compared to those who did not experience violence. The lower odds value was observed for women ever experiencing emotional violence (aOR = 1.17; 95% CI: 0.79, 1.74). Women ever experiencing any kinds of IPV was significantly associated with abortion (aOR = 1.38; 95% CI: 1.01, 1.90) than those who have not experienced violence (Table 5).

Our study also found that women whose husband had a history of alcohol use were more likely to have abortion (*aOR* = 1.45; 95% CI:1.09, 1.93) compared to those without the history of alcohol use (Supplementary Table 1).

## 4. Discussion

Despite numerous reproductive health care policies and programs in place, the prevalence of women who experience IPV in Nepal is high. This study found that women who experienced IPV are more likely to have abortion compared to those who did not experience violence.

The finding of higher abortion among women who experienced partner violence in our study is in agreement with previous research conducted in Nepal [8, 14, 41] India [43], Bangladesh [10, 34], and Pakistan [36]. The relationship between unwanted pregnancy and abortion has been well documented [34–36, 41]. A possible reason of higher abortion among women who experience IPV in this study may be that women in abusive relationships have low autonomy over their sexual lives; and therefore, can lead to unwanted pregnancy [34] and abortion [12].

Consistent with our finding, women with physical violence reported higher abortion rates in a number of studies conducted in India, [24, 44] Bangladesh [10, 34], and other African countries [45, 46]. In a violent spousal relationship, abortion can occur through various pathways. Firstly, women who experience partner violence may face pregnancy coercion which is often linked with lower contraception use and higher unintended pregnancy [47]. The association between unintended pregnancy and higher abortion has been previously established [24, 47]. Secondly, women in abusive relationships are more vulnerable to experience termination of pregnancy as they feel less prepared emotionally, socially, and financially to take care of a child into a violent setting which may influence women's decision for abortion [34]. Thirdly, poor uptake of health services, especially delayed in seeking antenatal care and injuries during pregnancy, may result in abortion [48, 49]. Previous studies have shown that higher IPV and lower women's autonomy in decision making led to unintended pregnancies due to lack of access to emergency family planning services [24, 34, 50]. We found that women whose husband has a history of alcohol use were more likely to experience abortion than those with no alcohol use. A possible explanation could be that the husband's alcohol consumption may also lead to forced sex and lack of contraception use, resulting in unwanted pregnancies and abortion [20].

Despite the positive association, the relationship between sexual violence and abortion in this study was not statistically significant. Consistent with the findings of this study, the previous study from Bangladesh reported no association between sexual violence and abortion [34], and a similar study conducted in the USA revealed lower rates of abortion among women who experienced sexual violence [51]. A possible reason could be a high stigma associated with talking about sex in Nepalese society as women might feel embarrassed to reveal their experience of sexual violence [52]. Generally, there is a perceived notion that forceful sexual acts by intimate partner do not constitute violence in Nepal [53], and stigma may have led such incident [34]. A multicountry study conducted by the WHO reported the higher acceptance of sexual violence compared to physical violence [1, 21]; and studies conducted in Nepal [54, 55] stated the lower rate of sexual violence due to underreporting. Unlike our study, the positive association between sexual violence and abortion has been reported in India and Bangladesh [10, 22]. A study from Nepal reported an association with sexual violence and unintended pregnancy [41], which may have occurred due to male dominance in a sexual decision, limited women's control over fertility, and limited access and use of contraceptives [41].

The emotional violence in the present study was not significantly associated with abortion. The findings are similar to those from studies conducted in Bangladesh [10] and Cameroon [45]. The possible explanation could be due to a lack of awareness about emotional violence and poor reporting [54]. Contrary to this study, a study conducted in Pakistan [36] and Kenya [56] reported a stronger association between emotional violence and abortion. The possible reason for higher abortion among women who reported emotional violence in Pakistani and Kenyan studies could be due to poor partner communication regarding reproductive health issues and reproductive inequality in violent relationships which may undermine the use of contraceptives [12, 57].

The current findings underline the importance to adopt effective strategies for married women and their partner in Nepal to reduce IPV and abortion. The Government of Nepal has focused on reducing abortion associated with maternal deaths through improving access to family planning methods. However, the use of such methods may be complex for women in a violent relationship. So, the appropriate measures through existing family planning program may be beneficial [7, 58–60]. Long-acting reversible contraceptive (LARCs) methods can reduce the risk of unintended pregnancy. Moreover, it should be assured that contraception should be easily accessible and widely available through government health facilities that will encourage women to use, even when they have lower autonomy by partner violence [7, 41].

The strengths of the present study include firstly, the analysis was based on pooled data of two consecutive nationally representative surveys conducted in 2011 and 2016 in Nepal, which maximised the sample size and improved the generalizability of the results to the wider population. Secondly, the NDHS used a pretested and well-designed questionnaire along with trained interviewers for data collection with good reliability. Thirdly, to our knowledge, this is the first national-level study to examine the association between IPV and abortion in Nepal, and the findings of this study may have some implications for countries of similar culture within South Asia. However, this study is limited by a number of reasons. First, it was a survey design which does not provide us with inferences regarding causality. Second, the underreporting of sexual violence due to more sensitive topic may have biased the results. Third, the important covariates such as antenatal care, knowledge about contraceptives that previous studies [48, 61] found as an important predictor of abortion could not be used in this study due to data unavailability, and inclusion of these variables would have changed the results of the final regression model.

### 4.1. Policy and Programmatic Implications

This study has policy and program implications. The current study indicates the urgency to safeguard women from intimate partner violence and prevent abortion. The Nepalese health system could address this issue through violence screening and provision of counselling support [10, 45]. Integrating IPV screening through antenatal care visits or family planning services at the health facility could be important steps to identify and offer counselling services to those who have experienced IPV [24]. Furthermore, the government should implement comprehensive reproductive health interventions to reduce risk factors for unintended pregnancy and address the need of the women who are suffering from violence [10]. Raising awareness among married women and providing social support would also help to better equip and prepared to deal with such challenging circumstances [62]. The engagement of intimate partner (mainly those with alcohol drinking habit) in education and counselling program is crucial. In addition, the existing government law for protecting women from abuse and violence must be strictly implemented.

## 5. Conclusion

Intimate partner violence was associated with abortion among women in Nepal. This study concludes that pregnant women who experience IPV in Nepal are more predisposed to have abortion. It is imperative that public health intervention, including the early screening, identification, and timely management of IPV. Therefore, along with the provision of a range of contraceptives, maternal health service sites need to be strengthened to cater assessment, counselling, and referral services for IPV to reduce unintended pregnancy that ends in abortion in Nepal.

## Figures and Tables

**Table 1:** Married women aged 15-49 years who have had at least one pregnancy five years preceding the survey and whoever experienced intimate partner violence in Nepal (2011-2016).

Types of IPV	Specific attributes of violence	Frequencies (*N* = 2978)	% (95% CI)
Physical	Pushed her, shook her, or threw something at her	403	13.6 (11.2, 16.2)
	Slapped her	580	19.5 (15.8, 23.8)
	Twisted arm or pulled her hair	252	8.5 (6.9, 10.3)
	Punched her with his fist or with something that could hurt her	222	7.4 (6.0, 9.2)
	Kicked her, dragged her, or beat her up	264	8.9 (6.8, 11.4)
	Tried to choke her or burn	74	2.5 (1.8, 3.4)
	Threatened her or attacked her with a knife or other weapon	60	2.0 (1.6, 2.6)
	Any physical violence	662	22.2(18.3, 26.7)
	Any less severe physical violence	652	21.8 (18.1, 26.2)
	Any severe physical violence	281	9.4 (7.4, 11.9)

Sexual	Physically forced into unwanted sex	316	10.6 (8.1, 13.7)
	Physically forced into other unwanted sexual acts	102	3.4 (2.3, 4.9)
	Physically forced to perform sexual acts respondent did not want	66	2.2 (2.7, 7.1)
	Any sexual violence	334	(8.8, 14.3)

Emotional	Ever been humiliated by husband	233	7.8 (6.3, 9.7)
	Ever been threatened with harm by husband	127	4.3 (3.4, 5.4)
	Ever been insulted or made to feel bad by husband	311	10.4 (8.7, 12.5)
	Any emotional violence	419	14.0 (12.1, 16.2)

Any forms of violence	Any (including, sexual, emotional, less severe, or severe violence)	839	28.2 (24.0, 32.8)

**Table 2:** Number of violence and corresponding prevalence of abortion with 95% confidence interval, NDHS (2011-2016).

Types of intimate partner violence (*N* = 2978)	Women with violence (multiple response) (*n* = 839) (%)	% abortion (95% CI)
Any physical violence	662 (22.2)	10.5 (6.9, 15.5)
Any sexual violence	334 (11.2)	11.5 (6.6, 19.4)
Any emotional violence	419 (14.1)	10.6 (6.1, 17.9)
Any less severe physical violence	652 (21.9)	10.3 (6.8, 15.3)
Any severe physical violence	281 (9.4)	11.7 (7.8, 17.3)
Any of the above violence	839 (28.2)	10.2 (6.9, 14.8)

**Table 3:** Characteristics of the study participants who experienced intimate partner violence in Nepal (2011-2016).

Characteristics	Categories	Total women (*N* = 2978)	%	Violence-experienced women (*n* = 839)	% of violence
Ecological region	Mountain	272	9.1	61	7.3
	Hill	1368	46.0	280	33.4
	Terai	1338	44.9	498	59.3

Household wealth status (quintiles)	Poorest	711	23.9	206	24.6
	Poorer	583	19.6	189	22.5
	Middle	600	20.2	210	25.1
	Richer	584	19.6	152	18.2
	Richest	500	16.8	82	9.8

Women's education	None	1112	37.3	443	52.8
	Primary	572	19.2	164	19.5
	Secondary or higher	1294	43.5	232	27.7

Husband's education	None	489	16.4	232	27.7
	Primary	666	22.4	233	27.7
	Secondary or higher	1823	61.2	374	44.6

Ethnicity	Brahman/Chhetri	999	33.6	187	22.3
	Janajati	975	32.7	245	29.2
	Dalit	492	16.5	177	21.1
	Other (e.g., Muslims)	512	17.2	230	27.5

Age group in years	15-24	1181	39.7	308	36.7
	25-34	1421	47.7	407	48.5
	35-49	376	12.6	124	14.7

Living children	0	87	2.9	22	2.6
	1	1026	34.4	209	24.9
	2	905	30.4	231	27.5
	≥3	960	32.3	377	45.0

Women decision making on own health care	Involvement of husband or family member	1254	42.1	391	46.6
	Involvement of husband and women	1020	34.2	253	30.2
	Involvement of respondent alone	704	23.7	195	23.2

Husband's alcohol drinking habit	No	1550	52.0	325	38.8
	Yes	1428	48.0	514	61.2

**Table 4:** Women with and without IPV and abortion in Nepal (2011-2016).

Variables	Categories	Total abortion (*N* = 292)	Women without IPV and abortion (*n* = 206)		Women with IPV and abortion (*n* = 86)		*p* value
			Number	%	Number	%	
Ecological region	Mountain	24	18	8.8	6	6.9	0.067
	Hill	140	107	51.8	34	39.5	
	Terai	128	81	39.4	46	53.4	

Household wealth status (quintiles)	Poorest	48	39	18.9	9	10.1	0.367
	Poorer	41	25	12.3	16	18.6	
	Middle	51	35	17.1	16	18.3	
	Richer	57	39	19.05	18	21.4	
	Richest	95	68	32.7	27	31.6	

Women's education	None	76	46	22.1	30	35.2	0.112
	Primary	66	45	21.9	21	24.0	
	Secondary or higher	150	115	56.0	35	40.8	

Husband's education	None	22	10	5.0	12	13.5	0.023^**†**^
	Primary	59	35	16.8	24	28.0	
	Secondary or higher	211	161	78.2	50	58.5	

Ethnicity	Brahman/Chhetri	145	110	53.3	35	41.0	0.101
	Janajati	83	58	28.1	25	28.7	
	Dalit	37	25	12.2	12	13.7	
	Other (e.g., Muslims)	27	13	6.5	14	16.5	

Age group	15-24	55	40	19.3	15	17.4	0.272
	25-34	157	118	57.1	40	46.2	
	35-49	80	48	23.6	31	36.4	

Living children	0	14	10	4.9	4	4.5	0.029^**†**^
	1	66	53	25.8	12	14.7	
	2	107	81	39.3	27	31.1	
	3 or more	105	62	29.9	43	49.7	

Women decision making on own health care	Involvement of husband or family member	86	63	30.5	23	26.8	0.494
	Involvement of husband and women	115	77	37.6	38	44.7	
	Involvement of respondent alone	91	66	31.9	25	28.5	

Husband's alcohol drinking habit	No	136	104	50.4	32	37.7	0.103
	Yes	156	102	49.6	54	62.3	

*p* value obtained from the chi-squared test. ^**†**^Significant at *p* < 0.05.

**Table 5:** Logistic regression analysis of the association between abortion and various forms of IPV in Nepal (2011-2016).

Types of IPV^#^	Categories	Crude OR (95% CI)	*p* value	Adjusted OR (95% CI)	*p* value
Any physical IPV (*N* = 662)	No	1.00		1.00	0.014^**†**^
	Yes	1.25 (0.91, 1.72)	0.170	1.54 (1.09, 2.19)	
Any sexual IPV (*N* = 334)	No	1.00		1.00	0.136
	Yes	1.17 (0.79, 1.75)	0.431	1.39 (0.90, 2.13)	
Any emotional IPV (*N* = 419)	No	1.00		1.00	0.435
	Yes	1.17 (0.81, 1.70)	0.405	1.17 (0.79, 1.74)	
Any less severe physical IPV (*N* = 652)	No	1.00		1.00	0.025^**†**^
	Yes	1.21 (0.88, 1.66)	0.252	1.50 (1.05, 2.13)	
Any severe physical IPV (*N* = 281)	No	1.00		1.00	0.026^**†**^
	Yes	1.36 (0.89, 2.09)	0.235	1.68 (1.06, 2.64)	
Any of the above (*N* = 839)	No	1.00		1.00	0.011^**†**^
	Yes	1.16 (0.86, 1.56)	0.324	1.38 (1.01, 1.90)	

*p* value obtained from the chi-squared test. Each type of violence was comprised of an independent regression model. ^**†**^Significant at *p* < 0.05. ^#^Adjusted for the following covariates: survey year, ecological region, household wealth, woman's education, husband's education, ethnicity, age, and number of living children, women's decision making on own health care, and husband's alcohol drinking habit.

## Data Availability

Data used in this study are publicly available secondary data obtained from the DHS (https://dhsprogram.com/data/available-datasets.cfm) program.

## Supplementary Materials

Supplementary Materials Supporting Table 1: logistic regression analysis of the association of abortion and any forms of violence in Nepal (2011-2016).

## References

[1] Hall M., Chappell L. C., Parnell B. L., Seed P. T., Bewley S. (2014). Associations between intimate partner violence and termination of pregnancy: a systematic review and meta-analysis. *PLoS Med*.

[2] Shai N., Pradhan G. D., Chirwa E., Shrestha R., Adhikari A., Kerr-Wilson A. (2019). Factors associated with IPV victimisation of women and perpetration by men in migrant communities of Nepal. *PLoS One*.

[3] Danitz S. B., Stirman S. W., Grillo A. R. (2019). When user-centered design meets implementation science: integrating provider perspectives in the development of an intimate partner violence intervention for women treated in the United States’ largest integrated healthcare system. *BMC Women's Health*.

[4] Kirk L., Terry S., Lokuge K., Watterson J. L. (2017). Effectiveness of secondary and tertiary prevention for violence against women in low and low-middle income countries: a systematic review. *BMC Public Health*.

[5] Wood S. L., Sommers M. S. (2011). Consequences of intimate partner violence on child witnesses : a systematic review of the literature. *Journal of Child and Adolescent Psychiatric Nursing*.

[6] Shamu S., Abrahams N., Temmerman M., Musekiwa A., Zarowsky C. (2011). A systematic review of African studies on intimate partner violence against pregnant women: prevalence and risk factors. *PLoS One*.

[7] Gilles K. *Intimate partner violence and family planning: opportunities for action*.

[8] Deuba K., Mainali A., Alvesson H. M., Karki D. K. (2016). Experience of intimate partner violence among young pregnant women in urban slums of Kathmandu Valley, Nepal: a qualitative study. *BMC Women's Health*.

[9] Puri M., Tamang J., Shah I. (2011). Suffering in silence: consequences of sexual violence within marriage among young women in Nepal. *BMC Public Health*.

[10] Rahman M. (2015). Intimate partner violence and termination of pregnancy: a cross-sectional study of married Bangladeshi women. *Reproductive Health*.

[11] da C Azevedo A. C., de Araújo T. V. B., Valongueiro S., Ludermir A. B. (2013). Intimate partner violence and unintended pregnancy: prevalence and associated factors. *Cadernos de Saúde Pública*.

[12] Pallitto C. C., García-Moreno C., Jansen H. A. F. M. (2013). Intimate partner violence, abortion, and unintended pregnancy: results from the WHO multi-country study on women’s health and domestic violence. *International Journal of Gynecology & Obstetrics*.

[13] World Health Organization (2013). Global and regional estimates of violence against women: prevalence and health effects of intimate partner violence and non-partner sexual violence. https://www.who.int/reproductivehealth/publications/violence/9789241564625/en/.

[14] Lamichhane P., Puri M., Tamang J., Dulal B. (2011). Women’s status and violence against young married women in rural Nepal. *BMC Women's Health*.

[15] Pun K. D., Infanti J. J., Koju R., Schei B., Darj E., on behalf of the ADVANCE Study Group (2016). Community perceptions on domestic violence against pregnant women in Nepal: a qualitative study. *Global Health Action*.

[16] Office of the United Nations High Commissioner for Human Rights The Nepal Conflict Report. http://www.ohchr.org/EN/Countries/AsiaRegion/Pages/NepalConflictReport.aspx.

[17] Ministry of Health and Population (MOHP) [Nepal] New ERA and ICF International Inc (2017). *Nepal Demographic and Health Survey 2016*.

[18] Yoshikawa K., Shakya T. M., Poudel K. C., Jimba M. (2014). Acceptance of wife beating and its association with physical violence towards women in Nepal: a cross-sectional study using couple’s data. *PLoS One*.

[19] Ministry of Health and Population (MOHP) [Nepal] New ERA and ICF International Inc (2012). *Nepal Demographic and Health Survey 2011, Kathmandu Nepal: Ministry of Health and Population*.

[20] Silwal P. Violence during pregnancy among young married women in Nepal. http://safpj.co.za/index.php/safpj/article/viewFile/1261/1588.

[21] Garcia-Moreno C., Jansen H. A. F. M., Ellsberg M., Heise L., Watts C. H. (2006). Prevalence of intimate partner violence: findings from the WHO multi-country study on women’s health and domestic violence. *The Lancet*.

[22] Silverman J. G., Raj A. (2014). Intimate partner violence and reproductive coercion: global barriers to women’s reproductive control. *PLoS Medicine*.

[23] Kaye D. K., Mirembe F. M., Bantebya G., Johansson A., Ekstrom A. M. (2006). Domestic violence as risk factor for unwanted pregnancy and induced abortion in Mulago Hospital, Kampala, Uganda. *Tropical Medicine and International Health*.

[24] Stephenson R., Jadhav A., Winter A., Hindin M. (2016). Domestic violence and abortion among rural women in four Indian states. *Violence Against Women*.

[25] Chibber K. S., Krishnan S. (2011). Confronting intimate partner violence: a global health priority. *Mount Sinai Journal of Medicine: A Journal of Translational and Personalized Medicine*.

[26] Rahman M., Sasagawa T., Fujii R., Tomizawa H., Makinoda S. (2012). Intimate partner violence and unintended pregnancy among Bangladeshi women. *Journal of Interpersonal Violence*.

[27] Tsui A. O., McDonald-Mosley R., Burke A. E. (2010). Family planning and the burden of unintended pregnancies. *Epidemiologic Reviews*.

[28] Liu J., Wu S., Xu J., Temmerman M., Zhang W.-H., The INPAC Group (2019). Is repeat abortion a public health problem among chinese adolescents? A cross-sectional survey in 30 provinces. *International Journal of Environmental Research and Public Health*.

[29] Institute G *Abort Worldwide 2017 Uneven Prog Unequal Access*.

[30] Jones R. K., Darroch J. E., Henshaw S. K. (2002). Contraceptive use among U.S. women having abortions in 2000-2001. *Perspectives on Sexual and Reproductive Health*.

[31] Yogi A., Prakash K. C., Neupane S. (2018). Prevalence and factors associated with abortion and unsafe abortion in Nepal: a nationwide cross-sectional study. *BMC Pregnancy and Childbirth*.

[32] Tamang A., Tuladhar S., Tamang J., Ganatra B., Dulal B. (2012). Factors associated with choice of medical or surgical abortion among women in Nepal. *International Journal of Gynecology & Obstetrics*.

[33] Thapa S., Neupane S. (2013). Risk factors for repeat abortion in Nepal. *International Journal of Gynecology & Obstetrics*.

[34] Silverman J. G., Gupta J., Decker M. R., Kapur N., Raj A. (2007). Intimate partner violence and unwanted pregnancy, miscarriage, induced abortion, and stillbirth among a national sample of Bangladeshi women. *BJOG: An International Journal of Obstetrics & Gynaecology*.

[35] Begum S., Dwivedi S. N., Pandey A., Mittal S. (2010). Association between domestic violence and unintended pregnancies in India: findings from the National Family Health Survey-2 data. *National Medical Journal of India*.

[36] Zakar R., Nasrullah M., Zakar M. Z., Ali H. (2016). The association of intimate partner violence with unintended pregnancy and pregnancy loss in Pakistan. *International Journal of Gynecology & Obstetrics*.

[37] Numan M. (2015). Sexual behaviors and sexual differentiation. *Neurobiology of Social Behavior*.

[38] Khatri R. B., Poudel S., Ghimire P. R. (2019). Factors associated with unsafe abortion practices in Nepal: pooled analysis of the 2011 and 2016 Nepal Demographic and Health Surveys. *PLoS ONE*.

[39] Solanke B. L. (2014). Association between intimate partner violence and utilisation of maternal health services in Nigeria. *African Population Studies*.

[40] Shahabuddin A. S. M., De Brouwere V., Adhikari R., Delamou A., Bardaj A., Delvaux T. (2017). Determinants of institutional delivery among young married women in Nepal: evidence from the Nepal Demographic and Health Survey, 2011. *BMJ Open*.

[41] Acharya K., Paudel Y. R., Silwal P. (2019). Sexual violence as a predictor of unintended pregnancy among married young women: evidence from the 2016 Nepal demographic and health survey. *BMC Pregnancy and Childbirth*.

[42] Joshi C., Torvaldsen S., Hodgson R., Hayen A. (2014). Factors associated with the use and quality of antenatal care in Nepal : a population-based study using the demographic and health survey data. *BMC Pregnancy and Childbirth*.

[43] Dhar D., McDougal L., Hay K. (2018). Associations between intimate partner violence and reproductive and maternal health outcomes in Bihar, India: a cross-sectional study. *Reproductive Health*.

[44] Lee-Rife S. M. (2010). Women’s empowerment and reproductive experiences over the lifecourse. *Social Science & Medicine*.

[45] Alio A. P., Salihu H. M., Nana P. N., Clayton H. B., Mbah A. K., Marty P. J. (2011). Association between intimate partner violence and induced abortion in Cameroon. *International Journal of Gynecology & Obstetrics*.

[46] Alio A. P., Nana P. N., Salihu H. M. (2009). Spousal violence and potentially preventable single and recurrent spontaneous fetal loss in an African setting: cross-sectional study. *The Lancet*.

[47] Miller E., Decker M. R., McCauley H. L. (2010). Pregnancy coercion, intimate partner violence and unintended pregnancy. *Contraception*.

[48] Tesfaye G., Loxton D., Chojenta C., Semahegn A., Smith R. (2017). Delayed initiation of antenatal care and associated factors in Ethiopia: a systematic review and meta-analysis. *Reproductive Health*.

[49] Sambisa W., Angeles G., Lance P. M., Naved R. T., Curtis S. L. (2010). Physical and sexual abuse of wives in urban Bangladesh: husbands reports. *Studies in Family Planning*.

[50] Bellizzi S., Mannava P., Nagai M., Sobel H. L. (2020). Reasons for discontinuation of contraception among women with a current unintended pregnancy in 36 low and middle-income countries. *Contraception*.

[51] Ely G. E., Murshid N. S. (2018). The relationship between partner violence and number of abortions in a national sample of abortion patients. *Violence and Victims*.

[52] Office of the Prime Minister and Council of Ministers, Singha Durbar, Kathmandu Nepal A Study on Gender-Based Violence Conducted in Selected Rural Districts of Nepal. https://asiafoundation.org/resources/pdfs/OPMCMGECUGBVResearchFinal.pdf.

[53] Ghimire A., Samuels F. (2017). *Understanding Intimate Partner Violence in Nepal: Prevalence, Drivers and Challenges*.

[54] Hawkes S. (2013). *Tracking Cases of Gender-Based Violence in Nepal: Individual, Institutional, Legal and Policy Analyses*.

[55] Hirachan N., Limbu D. (2017). An overview of sexual assault cases in Nepal. *Journal of Gandaki Medical College-Nepal*.

[56] Emenike E., Lawoko S., Dalal K. (2008). Intimate partner violence and reproductive health of women in Kenya. *International Nursing Review*.

[57] Cripe S. M., Sanchez S. E., Perales M. T., Lam N., Garcia P., Williams M. A. (2008). Association of intimate partner physical and sexual violence with unintended pregnancy among pregnant women in Peru. *International Journal of Gynecology & Obstetrics*.

[58] Raj A., McDougal L., Reed E., Silverman J. G. (2015). Associations of marital violence with different forms of contraception: cross-sectional findings from South Asia. *International Journal of Gynecology & Obstetrics*.

[59] Raj A., McDougal L. (2015). Associations of intimate partner violence with unintended pregnancy and pre-pregnancy contraceptive use in South Asia. *Contraception*.

[60] Chen G. L., Silverman J. G., Dixit A. (2020). A cross-sectional analysis of intimate partner violence and family planning use in rural India. *EClinicalMedicine*.

[61] Ankomah A., Klutsey E. (2014). Factors associated with induced abortion at selected hospitals in the Volta region, Ghana. *International Journal of Women's Health*.

[62] Jewkes R. (2002). Intimate partner violence : causes and prevention. *The Lancet*.

