# Supplementary material for: Association between Intimate Partner Violence and Abortion in Nepal: A Pooled Analysis of Nepal Demographic and Health Surveys (2011 and 2016)
Source: Biomed Res Int. 2020 Aug 31;2020:5487164. doi: 10.1155/2020/5487164 (PMC7481936; doi:10.1155/2020/5487164)
Supplement: Supplementary Materials — Supporting Table 1: logistic regression analysis of the association of abortion and any forms of violence in Nepal (2011-2016). [file 5487164.f1.docx]

Supporting table 1**.** Logistic regression analysis of the association of abortion and any forms of violence in Nepal (2011-2016).

| **Variables** | **Categories** | **Adjusted OR (95% CI)** | **p-value** |
| --- | --- | --- | --- |
| Survey Year | 2011 | Reference category |  |
|  | 2016 | 1.45(1.07,1.97) | 0.015 |
| Household wealth (quintiles) | First (poorest) | Reference category |  |
|  | Second (poorer) | 1.25 (0.77,2.05) | 0.363 |
|  | Third (middle) | 1.73 (1.03,2.88) | 0.038 |
|  | Fourth (richer) | 1.84 (1.08,3.13) | 0.026 |
|  | Fifth (richest) | 2.90 (1.67,5.02) | <0.001 |
| Women’s education | None | Reference category |  |
|  | Primary | 2.09 (1.39,3.14) | <0.001 |
|  | Secondary or higher | 1.35 (0.87,2.10) | 0.174 |
| Husbands education | None | Reference category |  |
|  | Primary | 1.80 (1.03,3.17) | 0.040 |
|  | Secondary or higher | 2.20 (1.25,3.87) | 0.006 |
| Ethnicity | Brahman/Chhetri | Reference category |  |
|  | Janajati | 0.50 (0.36,0.71) | <0.001 |
|  | Dalit | 0.67 (0.43,1.06) | 0.087 |
|  | Other (e.g., Muslim) | 0.36 (0.21,0.62) | <0.001 |
| Age group | 15-24 | Reference category |  |
|  | 25-34 | 2.15 (1.47,3.12) | <0.001 |
|  | 35-49 | 6.39 (3.87,10.56) | <0.001 |
| Living children | 0 | Reference category |  |
|  | 1 | 0.34 (0.17,0.69) | 0.003 |
|  | 2 | 0.53 (0.27,1.07) | 0.075 |
|  | 3 or more | 0.50 (0.24,1.06) | 0.073 |
| Husband’s habits of alcohol use | No | Reference category |  |
|  | Yes | 1.45 (1.09,1.93) | 0.011 |

Table 1 shows the association between abortion and any forms of IPV. It was revealed that women from the fifth richest wealth quintile (adjusted Odds Ratio=2.90; 95% CI: 1.67, 5.02) were more likely to have abortion who experienced any violence compare to other wealth groups. Women’s education as primary (aOR=2.09; 95% CI: 1.39, 3.14) and husband’s education secondary or higher (aOR =2.20; 95% CI: 1.25, 3.87) were more likely to have abortion who experienced any violence as compared to the reference group. Additionally, women aged between 35-49 years (aOR =6.39; 95% CI: 3.87, 10.56) had six-fold higher odds of having abortion who experienced any violence as compared to other age groups. Moreover, the women whose husband had a history of alcohol use and experienced any forms of violence were more likely to have abortion (aOR =1.45; 95% CI: 1.09, 1.93) than those who didn’t experience violence [Table 1].
